# Functions of Phenylalanine Residues within the β-Barrel Stem of the Anthrax Toxin Pore

**DOI:** 10.1371/journal.pone.0006280

**Published:** 2009-07-17

**Authors:** Jie Wang, Gregory Vernier, Audrey Fischer, R. John Collier

**Affiliations:** Department of Microbiology and Molecular Genetics, Harvard Medical School, Boston, Massachusetts, United States of America; Columbia University, United States of America

## Abstract

**Background:**

A key step of anthrax toxin action involves the formation of a protein-translocating pore within the endosomal membrane by the Protective Antigen (PA) moiety. Formation of this transmembrane pore by PA involves interaction of the seven 2β2–2β3 loops of the heptameric precursor to generate a 14-strand transmembrane β barrel.

**Methodology/Principal Findings:**

We examined the effects on pore formation, protein translocation, and cytotoxicity, of mutating two phenylalanines, F313 and F314, that lie at the tip the β barrel, and a third one, F324, that lies part way up the barrel.

**Conclusions/Significance:**

Our results show that the function of these phenylalanine residues is to mediate membrane insertion and formation of stable transmembrane channels. Unlike F427, a key luminal residue in the cap of the pore, F313, F314, and F324 do not directly affect protein translocation through the pore. Our findings add to our knowledge of structure-function relationships of a key virulence factor of the anthrax bacillus.

## Introduction

*Bacillus anthracis*, the causative agent of anthrax, produces two major virulence factors: a poly-D-glutamic acid capsule and anthrax toxin. The latter comprises three large monomeric proteins. The Lethal Factor (LF) and the Edema Factor (EF), are enzymes that act on substrates within the cytosol of mammalian cell. The third, Protective Antigen (PA; 83 kDa), is a receptor-binding and pore-forming protein that binds and transports LF and EF from the extracellular milieu of cells to the cytosol [1].

PA binds to a receptor at the cell surface and is proteolytically activated by a furin-family protease, yielding a 63 kDa form (PA_63_), which self-associates to form a ring-shaped heptamer, termed the prepore. The prepore binds 1–3 copies of EF and/or LF and undergoes receptor mediated endocytosis. Residence within the acidic environment of the endosome induces a conformational change in the PA moiety from the soluble prepore to a membrane-inserted, protein-conducting channel. The PA pore functions as a translocase, mediating unfolding and translocation of bound EF and LF across the membrane [2].

**Figure 1:**
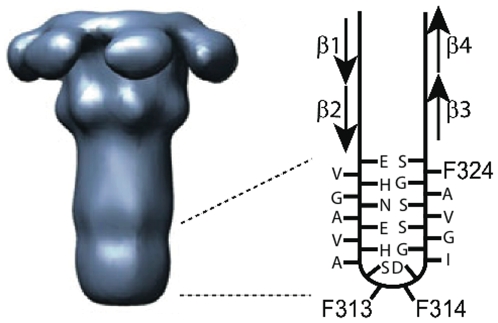
Locations of F313, F314, F324 and β strands 1–4 in the β barrel and the pore. The approximate boundaries of the membrane-spanning region are indicated by the dashed lines. The illustration of the pore structure (left), reconstructed from single-pore images obtained by electron microscopy, is modified from Figure 4 of reference [4]. The amino acid sequence of the ® hairpin is as follows: E(302)VHGNAEVHASFFDIGGSVSAGFS(325).

The PA pore is a mushroom-shaped structure, with a globular cap and a 100Å-long, 14-stranded, β-barrel stem formed from the seven 2β2–2β3 loops of the prepore (Fig. 1) [3], [4]. Within the cap there exists a solvent-exposed Phe residue, Phe427, that has been shown to play a crucial role in protein translocation through the pore [5]. The Phe427 side chains of the seven subunits form what has been called the Phe clamp and have been proposed to interact directly with the unfolded translocating polypeptide to form a seal against the passage of ions. This seal may function by preserving the transmembrane pH gradient within the pore, previously demonstrated to be the primary driving force for protein translocation [2].

At the turn region of the 2β2–2β3 β hairpins that form the stem of the pore there are two Phe residues, F313 and F314 (Fig. 1). Their locations suggest they form a hydrophobic tip of the 14-stranded β barrel that may aid in its insertion into the membrane and/or anchor the tip in the cap-distal leaflet of the bilayer. It is also conceivable, however, that one or both of these residues might face the lumen of the pore and play an active role in translocation, perhaps resembling that of Phe427. We therefore examined the functional consequences of mutating F313 and F314 to various other residues. We also examined the effect of mutating to Ala a third Phe residue in the β barrel proposed to lie on the endosomal leaflet of the membrane interface, F324.

## Materials and Methods

Proteins – Mutations were generated using site-directed mutagenesis (Stratagene) and confirmed by DNA sequencing. Recombinant PA, von Willebrand factor A (VWA) domain, residues 1–263 of LF (LF_N_), and LF_N_ fused to the catalytic domain of diphtheria toxin (LF_N_-DTA) were expressed and purified, and the heptameric PA prepore was prepared from the full length PA, as described [6]. Protein concentrations were estimated from A_280_ measurements with the following calculated molar extinction coefficients: PA_83_, 75,670 M^−1^ cm^−1^; PA_63_, 49,640 M^−1^ cm^−1^; VWA, 12,485 M^−1^ cm^−1^; LF_N_ 17,920 M^−1^ cm^−1^; LF_N_–DTA, 43,600 M^−1^ cm^−1^.

Liposome preparation – Phospholipid (1,2-dioleoyl-sn-glycero-3-phosphocholine) was dried under a nitrogen gas stream, followed by desiccation overnight. The lipid film was hydrated with 1 mL 10 mM HEPES, 100 mM KCl, pH 7.5 to a final concentration of 25 mg/ml, followed by three freeze-thaw cycles and extrusion 11 times through a 200 µm pore size polycarbonate filter (Whatman). The resulting liposomes were stored at 4°C. Immediately before the experiment, the liposomes were exchanged into 10 mM Tris, 100 mM NaCl, pH 8.5, using a G50 desalting column (GE Healthcare) and adjusted to a final concentration of 5 mg/ml.

K^+^ release assay – PA prepore (3 nM ) was incubated with 40 nM VWA domain (molar ratio of VWA domain to PA_63_ = 2) at room temperature for 15 min, and 20 µl of the sample was mixed with 200 µl liposomes. The mixture was then incubated 5 min and added to 5 ml working solution (50 mM sodium acetate, 100 mM NaCl, pH 5.0). K^+^ release was monitored by means of a K^+^-selective electrode [6]. The initial rate of K^+^ release (k) was calculated by linearly fitting the K^+^ release curve.

LF_N_ translocation across planar lipid bilayers – Planar lipid bilayers were generated as described [7]. The *cis* and *trans* compartments were bathed in 1 ml Universal Bilayer Buffer (UBB: 100 mM KCl, 1 mM EDTA, 10 mM each of potassium oxalate, potassium phosphate, 2-(N-morpholino)ethanesulfonic acid, and pH 5.5). Δψ (Δψ = ψ*_cis_*−ψ*_trans_*), the membrane potential, was set to +20 mV. PA_63_ heptamer (15 ng) was added to the *cis* compartment. Mutants that produced no channels after addition of1.5 µg PA_63_ heptamer were deemed to be devoid of channel-forming activity. For the mutants capable of forming channels, 0.1 nmol LF_N_ was added to the *cis* compartment after (PA_63_)_7_ channel formation stabilized. Unbound LF_N_ was removed by perfusion with 10 ml UBB at 2 ml/min. To initiate translocation, 7 µl 2 M KOH was added to the *trans* chamber to raise the pH to 7.2, and the change in current was monitored. Both compartments were stirred continuously throughout the experiments. The half time of translocation (*t*_1/2_) was calculated from sigmoidal fits of averaged normalized data.

Single-channel bilayer measurements - Single-channel measurements were obtained under conditions similar to macroscopic current recording with a 100 µm aperture membrane. The *cis* and *trans* compartments contained solutions of 1 M KCl, 10 mM 2-(N-morpholino)ethanesulfonic acid at pH 5.5, and current was meassured over a range of positive potentials (*cis*-positive). Increments of ∼0.1 ng of PA_63_ heptamer were added until a single channel was observed. Data were analyzed using Clampfit, version 10.0, software (Axon Instruments, Sunnyvale, CA), and Microsoft Excel. Analysis was performed on 10-s records. Single-channel conductance was calculated from Gaussian fits to current amplitude histograms. Experiments were repeated 3 times each.

Cellular LF_N_-DTA intoxication assay – The LF_N_-DTA intoxication assay was performed as described [8]. WT or mutant PA (0.1 µM–10^−9^ µM) was incubated with CHO-K1 cells in the presence of 0.1 µM LF_N_-DTA for 4 h at 37°C. The medium was then replaced with leucine-free F-12 medium supplemented with 1 µCi/ml ^3^H-leucine, and the cells were incubated at 37°C for 1 h. Translocation of the diphtheria toxin catalytic domain into the cells was measured by ^3^H-leucine incorporation into total cellular protein.

## Results

We used directed mutagenesis to replace F313 and F314 with various other amino acid residues and F324 with Ala. The mutant proteins were expressed in *Escherichia coli*, purified, and compared with wild-type PA in various assays. For cell-culture toxicity assays we used the purified monomeric proteins, and for assays in model membranes we used the heptameric prepore obtained by activating the monomers with trypsin and isolating the PA prepore by ion-exchange chromatography.

**Figure 2:**
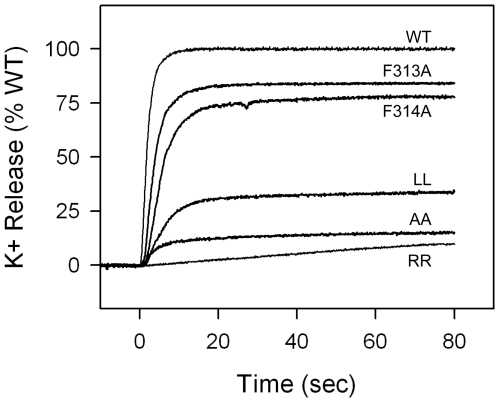
Effects of F313 and F314 mutations on PA permeabilization of membranes to K^+^. For clarity, only selected mutants are shown. The double Trp (WW) and double Tyr (YY) replacements had approximately the same kinetics of K^+^ release as the WT (FF). The kinetics of release by the double Leu (LL), double Ala (AA), and double Arg (RR), as well as the F313A and F314A mutants are also shown. The double His (HH), double Asp (DD), and the buffer control results were indistinguishable from those of RR.

To test the effects of mutations on pore formation in a model membrane, we assayed for K^+^ release from KCl-charged liposomes at pH 5.5. The prepore was complexed with the PA-binding VWA domain from anthrax toxin receptor ANTXR2. Binding of the VWA domain, besides approximating the *in vivo* state, improved the quality of data on the kinetics of K^+^ release by stabilizing the prepore and slowing its conversion to the pore conformation. As shown in Fig. 2 and Table 1, mutating both F313 and F314 to either Trp (WW) or Tyr (YY) had little effect on the kinetics of K^+^ release, whereas replacing them with Leu caused a two-fold inhibition of initial rate of release. Mutating both of these Phe residues to His (HH), Asp (DD) or Arg (RR), or deleting them (mutant S1) virtually ablated permeablization activity. Deletion of the entire ® strand segment proposed to insert into the membrane (residues 302–325) resulted in a mutant, the “loopless” mutant, that was incapable of permeablizing the membrane. Individual mutations of F313 or F314 to Ala caused 25–50% reduction in the initial rate of permeabilization, and the double Ala mutant reduced the initial rate ∼3-fold. Thus, efficient channel formation depended upon having hydrophobic residues at these positions, aromatic residues being the most active.

**Table 1:** Pore formation, LF_N_ translocation and LF_N_-DTA-dependent cytotoxicity mediated by F313X/F314X mutants.

Mutation	K^+^ release, initial rate (s^−1^)	LF_N_ translocation, *t*_1/2_ (s)	Cytotoxicity, EC_50_ (pM)
FF (wild-type)	24.4±4.0	12±.1	1.6±0.03
WW	22.1±1.9	12±.1	6.5±2.2
YY	23.5±4.7	13±.1	12±3.7
LL	11.2±1.4	12±.1	0.9±0.4
AA	7.3±3.3	*	10±2.2
F313A	17.5±1.0	12±.1	3.7±1.7
F314A	10.9±1.4	12±.1	1.7±0.3
HH	−	**	270±72
DD	−	**	590±230
RR	−	**	−
S1	−	**	2300±880
“Loopless”	−	**	−

− no activity relative to control.

* low pore forming activity; translocation not tested.

** no pores formed in planar bilayers.

Activity of these mutants in forming channels in planar phospholipid bilayers correlated well with activity observed in the K^+^ release assay (Table 1). Stable pores were found only with the double Trp, Tyr, and Leu mutants and the single F313A and F314A mutants. Few pores were seen with the double Ala mutant.

For a subset of the mutants we measured single-channel currents in planar bilayers. Wild-type PA elicited discrete channel openings with a single-channel conductance of 153±2 pS (in symmetric 1 M KCl). Single-channel conductance values for the double Leu (153±2 pS), double Ala (155±2 pS), and the single F313A (154±2 pS) mutants were indistinguishable from the wild-type (data not shown). Also, the probability of residing in the open state did not vary from the wild-type pore.

For each mutant that formed stable pores in planar bilayers, we examined its ability to translocate LF_N_, the N-terminal domain of LF, across the bilayer. Channels were formed in the membrane upon addition of prepore under an applied potential of +20 mV and symmetric pH 5.5. Subsequent addition of LF_N_ caused current blockage as this protein bound to the channel. After perfusion to remove excess LF_N_, the pH of the *trans* compartment was raised to pH 7.2, and translocation was monitored by the alleviation of channel block. The *t*_1/2_ of the translocation reaction deviated less than 10% from that of the wild type (∼12 s) (Table 1). These results imply that mutations at positions 313 and 314 that do not inhibit formation of stable channels are fully competent for protein translocation.

**Figure 3:**
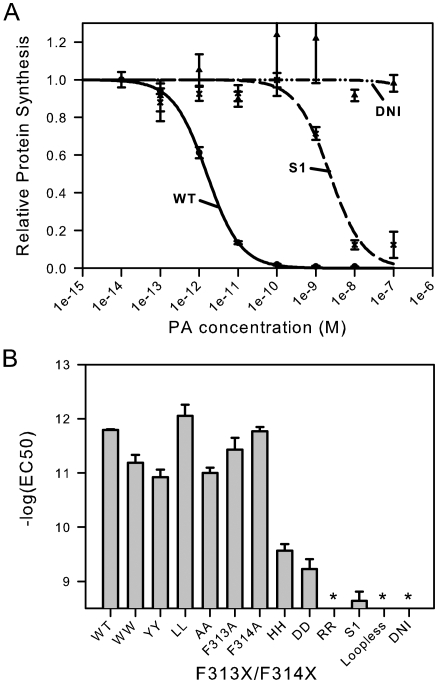
Cellular translocation assay of F313X/F314X mutants. (A) PA_83_ was titrated, mixed with a fixed concentration of LF_N_-DTA (100 pM), added to CHO-K1 cells, and incubated at 37°C for 4 hours. The level of protein synthesis was measured by detecting [^3^H] leucine incorporation and was expressed as fraction of that observed with PA-WT. Only WT, S1 (deletion of F313 and F314) and DNI (K397D, D425K) are shown. (B) EC_50_ for all F313X/F314X mutants as calculated from sigmoidal fits to cytotoxicity experiments.

To characterize these mutations in a cellular assay we measured the ability of the mutated proteins to transport a model intracellular effector protein, LF_N_-DTA, to the cytosolic compartment of CHO-K1. LF_N_-DTA is a fusion protein containing the N-terminal, prepore-binding domain of LF fused to DTA, the catalytic domain of diphtheria toxin. Delivery of LF_N_-DTA to the cytosol causes inhibition of protein synthesis, resulting in cell death at ∼1 pM PA and 0.1 ▒M LF_N_-DTA (Fig. 3). Removal of the 2β2–2β3 loop resulted in complete loss of cytoxicity, as did the incorporation of a dominant-negative double mutatation K397D, D425K (dubbed DNI, for dominant-negative inhibitor) [9]. The EC_50_ of all of the aromatic and/or non-polar mutants that retained efficient pore-forming activity deviated less than 10-fold from the wild-type value under the conditions of our assay (Table 1 and Fig. 3). Replacement of F313 and F314 with charged residues reduced LFn-DTA cytoxicity by at least 300 fold; mutation to two glycine residues resulted in complete ablation of cytotoxicity.

PA carrying the F324A mutation was tested for activity in the K^+^ release assay, in planar bilayers for translocation activity, and in cell culture for ability to mediate LF_N_-DTA-dependent cytotoxicity. No differences from wild-type PA were detected.

## Discussion

According to our current model of the membrane-inserted PA pore, F313 and F314 lie in the turn region of the 14-strand β-barrel stem, at or near the aqueous interface of the *trans* leaflet of the bilayer [3]. In porins and many other membrane proteins, aromatic residues densely populate the boundary between the nonpolar and interfacial regions of the bilayer and are thought to help anchor these proteins in the membrane [10], [11]. Crystal structures of β-barrel pore forming toxins like 〈 hemolysin and aerolysin have demonstrated that residues lining the *trans* leaflet of the bilayer in a rivet conformation must be hydrophobic in order to efficiently promote membrane insertion [12], [13]. Our results demonstrate that the PA is very sensitive to changes in the hydophobicity of the residues at the *trans* leaflet anchoring position, supporting the hypothesis that two Phe residues alone comprise the rivet [3]. We showed that hydrophobic residues at positions 313 and 314 function well; however hydrophobic aromatic residues are optimal.

While the His side chain contains six pi electrons capable of forming pi-stacking interactions it also becomes protonated at pH values below neutrality, and thus it is not surprising that mutation of F313 and F314 to His significantly attenuated PA channel insertion and intoxication. The model is consistent with the hypothesis that the side chains of both F313 and F314 serve to anchor the pore in the membrane. F313 and F314 may also facilitate insertion of the pore, presumably by generating a highly hydrophobic tip — a cluster of 14 Phe residues — that promotes partitioning into the bilayer.

The location of F324 in the primary structure suggests that its side chain occupies an analogous location in the interface region of the *cis* leaflet of the bilayer. Thus, the F324 residues on the *cis* leaflet and the F313 and F314 residues in the *trans* leaflet most likely form aromatic girdles analogous to those seen in many integral membrane proteins. We detected no effect of replacing F324 with Ala, indicating that stable pore formation is primarily dependent on the residues at the cytosolic leaflet rather than those at the endosomal leaflet.

The fact that single-channel conductance of the F313/F314 mutants examined remained unchanged from that of the wild-type protein in our experiments demonstrates that the passage of ions through the pore was unaffected by the side chains at these locations. Importantly, the half-time of translocation of LF_N_ under the influence of a transmembrane proton gradient did not vary from that of the wild-type protein. These findings are consistent with the notion that the side chains of F313 and F314 are embedded in the membrane, and do not affect passage of monovalent ions or proteins through the pore. The effects of mutating these Phe residues differed strongly from effects of mutating F427, where major changes were seen in both single-channel conductance and protein translocation.

The effects of F313/F314 mutations on delivery of LF_N_-DTA to the cytosol correlated well with the effects of these mutations on K^+^ release. Replacing these residues with charged amino acids had large effects on cytotoxicity, K^+^ release from liposomes, and formation of pores in planar bilayers, as would be predicted from the energetic barrier to membrane penetration by such residues. Deleting F313 and F314 presumably blocked membrane insertion and/or the stability of the pore in the membrane. Many explanations are possible for the smaller variations in activity seen among the other mutants, including, for example, effects on the kinetics of prepore-to-pore conversion resulting from altered side chain interactions with domains 2 and 4 surrounding the 2β2–2β3 loop in the prepore [6].
